# Utility and safety of nafamostat mesilate for anticoagulation in dogs

**DOI:** 10.1002/vms3.1002

**Published:** 2022-11-21

**Authors:** Noriko Isayama, Goki Matsumura, Yusuke Uchimura, Erika Maeda, Kenta Sasaki

**Affiliations:** ^1^ Department of Cardiology Ueno no Mori Animal Hospital Taito Tokyo Japan; ^2^ Department of Cardiovascular Surgery The Heart Institute of Japan Tokyo Women's Medical University Shinjyuku‐ku Tokyo Japan

**Keywords:** anticoagulants, cardiac surgery, cardiopulmonary bypass, mitral valve insufficiency

## Abstract

**Background:**

Surgical interventions are recommended for cases of advanced mitral regurgitation, however, limited facilities are available. The most prominent complication in such procedures is heparin‐derived bleeding. An alternative anticoagulant to heparin, nafamostat mesilate (NM), can reduce the occurrence of complications associated with heparin such as bleeding or shock.

**Objectives:**

This study aimed to evaluate the utility and safety of using NM during anaesthesia in canines.

**Methods:**

Six healthy adult Beagle dogs were anaesthetised, and NM was administered intravenously as a 10 mg/kg bolus dose over 5 min, followed by a continuous infusion of 10 mg/kg/h over 20 min. Blood tests and blood pressure measurements were performed at 0, 5, 25 and 55 min after NM administration.

**Results:**

Activated thromboplastin times at 0, 25 and 55 min were 13.0 ± 0.7 s, 106.7 ± 13.3 s and 28.2 ± 2.9 s, respectively, with a significant difference between 0 and 25 min (*p* < 0.01) only. No significant differences were observed in prothrombin time, antithrombin, fibrinogen and fibrin degradation product concentrations between timepoints. Activated clotting times (ACTs) at 0, 5, 25 and 55 min were 119.5 ± 9.6 s, 826.7 ± 78.6 s, 924.8 ± 42.4 s and 165.2 ± 13.5 s, respectively. Significant differences were observed between 0 and 5 min (*p* < 0.05) and between 0 and 25 min (*p* < 0.05). Blood pressure changes occurred in four dogs (66.7%). No other serious adverse effects were observed.

**Conclusions:**

ACT results indicated that NM use in anaesthetised healthy dogs was sufficient to obtain procedural anticoagulation with minimal adverse effects. However, these preliminary data require validation in further studies on cardiopulmonary bypass surgery.

## Graphical abstract

This study aimed to evaluate the utility and safety of using nafamostat mesilate during anesthesia in canines. Blood tests and blood pressure measurements were performed at 0, 5, 25, and 55 min after nafamostat mesilate administration. The study found activated clotting time prolongation was sufficient with nafamostat mesilate use, indicating its safety during anesthesia in healthy dogs.

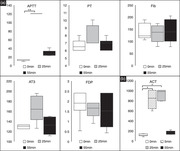

## 1 INTRODUCTION

Open heart surgery using cardiopulmonary bypass (CPB) is a treatment method for canine heart disease (Keene et al., 2019). This is a high‐risk procedure with the potential for several complications and, currently, it is performed in just a few facilities worldwide. Administration of anticoagulant drugs is necessary during CPB, and heparin is commonly used in humans and dogs (Bull et al., 1975; Uechi, 2012). The effects of heparin need to be reversed using protamine after CPB to control bleeding. However, severe shock can be caused by protamine in dogs, and protamine also has anticoagulant effects (Marin‐Neto et al., 1979). Haemodilution after CPB and persistent heparin effects pose challenges for haemorrhage control in humans (Ball et al., 2016). These challenges make it difficult to conduct open‐heart surgery in dogs; thus, a limited number of facilities can perform this surgery. Low‐molecular‐weight heparin (LMWH), argatroban (ALG) and nafamostat mesilate (NM) are used as substitutes for heparin during CPB in patients with heparin‐induced immune thrombocytopenia (Agarwal et al., 2012; Kawamoto et al., 2020). To control bleeding after incising the heart, the anticoagulation effect should not persist beyond discontinuation of CPB during the operation. Therefore, studies have aimed to evaluate the short‐term effects of NM in comparison to those of LMWH and ALG.

NM is used for pancreatitis in humans and dogs (Yamamoto et al., 2016; Lukaszyk et al., 1992), and because of its anticoagulant properties, it has been clinically used during CPB in humans for whom heparin cannot be used, such as in heparin‐induced immune thrombocytopenia cases (Kawamoto et al., 2020; Murase et al., 1993). NM is metabolised by carboxylesterase in liver cells and blood. The half‐life of NM in dogs and rats has been reported to be 1 and 8 min, respectively, in the bloodstream, and its anticoagulant effect is quickly lost (Okajima et al., 1995; Aoyama et al., 1984). Other studies have described NM having a half‐life of 8 min (Aoyama et al., 1985) and 16 min (Aoyama, 1984) in dogs. Due to its short duration of action, it assists in reducing further complications, such as bleeding or protamine shock, and its anticoagulant effect disappears without the use of antagonists.

This study evaluated the utility and safety of NM in anaesthetised dogs. The study aimed to establish whether NM could help control bleeding through the measurement of several blood clotting parameters. It was hypothesised that NM may be a substitute for heparin with a superior safety profile compared to heparin.

## 2 MATERIALS AND METHODS

### 2.1 Animal experiments

The experimental procedures were conducted using six healthy adult female Beagle dogs (age, 24 ± 7 months; NARC Co., Tomisato, Japan). The dogs were positioned laterally. Indirect BP was measured using a non‐invasive oscillometric monitor (BP100D, FUKUDA ME, Tokyo, Japan; pet MAP, Ramsey Medical, Inc., Tampa, FL, USA). The blood pressure monitoring cuff was placed directly around the tail, minimising the vertical distance between the cuff site and the heart and eliminating the need for pressure correction to account for the height difference. An appropriately sized cuff (inflatable bladder width approximately 0.3‒0.4 times the circumference of the measurement site) was applied.

An intravenous catheter was inserted into the cephalic vein, and butorfanol and midazolam were administered intravenously at a dose of 0.2 mg/kg each, followed by propofol at a dose of 6 mg/kg. The dogs were intubated after induction, and respiration was controlled using ventilation. Anaesthesia was maintained by continuous rate infusion (CRI) of propofol at a dose of 2–6 mg/kg/h. After stabilisation, defined as the point at which after intubation, fluctuations in heart rate, respiratory rate, and blood pressure had settled and remained unchanged for approximately 15 min, a baseline (0 min) blood draw was performed, and blood pressure (BP) and heart rate (HR) were measured. Another intravenous catheter was inserted in the contralateral cephalic vein for NM administration. A 10 mg/kg NM (FUTHAN50 INJ, Nichi‐Iko Pharmaceutical Co., Ltd, Tokyo, Japan) bolus dose was administered via this catheter over 5 min, and the activated clotting time (ACT) was measured after the bolus dose of NM administration was completed (5 min). The administration of bolus NM was followed by 10 mg/kg/h CRI administration of NM for 20 min. After completion of this bolus of NM, blood was drawn, and BP and HR were measured again (25 min). Another 30 min was allowed for washout of NM, and blood was drawn again, and the BP and HR were measured (55 min). All samples were collected whilst the dogs received propofol, for ethical reasons. No other fluids or drugs were administered during anaesthesia.

### 2.2 Sample preparations

Blood samples [3.5 ml from each dog: 1.8 ml for coagulation studies, 0.5 ml for complete blood count (CBC), 0.2 ml for ACT, and 1.0 ml for biochemistry] were collected from the jugular vein and stored, respectively, in citrate‐buffer blood collection tubes to test for activated thromboplastin time (APTT), prothrombin time (PT), fibrinogen (Fib), antithrombin (AT) and fibrin degradation product (FDP) concentrations; in ACT cartridges for ACT measurements; in EDTA sample tubes to measure CBC; and in plain tubes for plasma biochemistry tests. The blood samples were acquired from fresh venepuncture on every occasion to avoid clotting or altered results from use of a venous catheter. Blood samples collected in citrate‐buffered blood collection tubes and plain tubes were centrifuged at 3000 rpm for 10 min at 4°C. ACT was measured using a cationic ACT cartridge (iSTAT, Abbott Laboratories, Abbott Park, IL, USA). CBC, plasma biochemistry and blood coagulation tests were performed in a commercial laboratory (FUJIFILM Monolith, Co., Ltd., Tokyo, Japan).

### 2.3 Data collection

The following data were collected and analysed: APTT, PT, Fib, AT and FDP concentrations; ACT, white blood cell (WBC) count, haematocrit count, platelet count and biochemical parameters (including total protein count, albumin level, blood glucose level, aspartate aminotransferase level, alanine transaminase level, concentrations of alkaline phosphatase, lactate dehydrogenase, blood urea nitrogen, creatinine, sodium, potassium and chloride); blood pressure (systolic, diastolic and mean); HR, mortality and adverse effects (diarrhoea and vomiting).

### 2.4 Statistical analysis

The distribution of study variables across different time points is described using the mean and standard error of the mean and illustrated graphically using boxplots. Although Shapiro–Wilk tests were performed to determine whether to use parametric or non‐parametric statistical tests, owing to the small sample size of the study, we automatically used non‐parametric repeated measures Friedman test with Dunn's multiple comparison tests to assess the differences in the distribution of these study variables across time points. Statistical significance was set at *p* < 0.05. Commercial software (InStat version 3.10, GraphPad Software Inc., San Diego, CA, USA) was used for statistical analyses.

### 2.5 Ethics

The authors confirm that the ethical policies of the journal, as noted on the journal's author guidelines page, have been adhered to, and the appropriate ethical review committee approval has been received (reference: AE20‐008‐3‐C; approval date: 5 October 2020). The US National Research Council's Guidelines for the Care and Use of Laboratory Animals were followed.

## 3 RESULTS

### 3.1 Haemostasis

**FIGURE 1:**
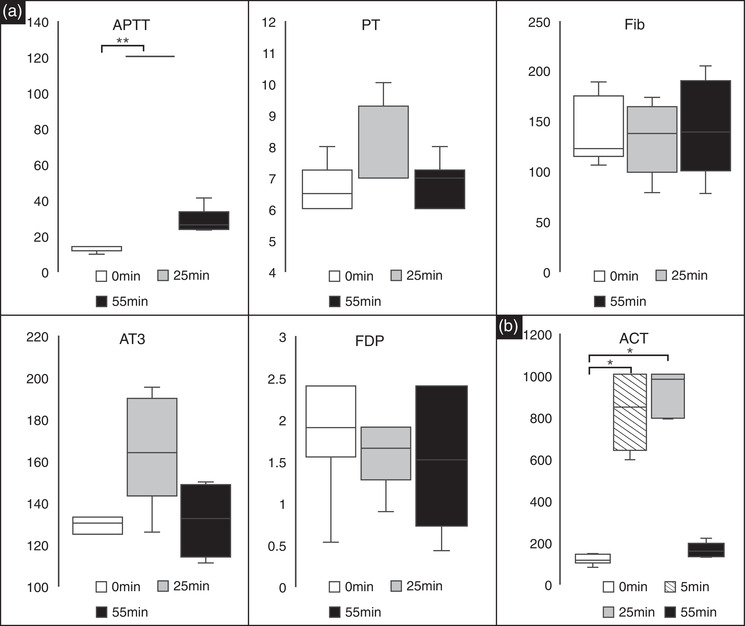
(a) Box and whisker plots for the blood clotting system analysis. Significant differences were observed in APTT (in seconds) between 0 versus 25 min (*p* < 0.01). There were no significant differences between any of the other time points. There were no significant differences in PT (seconds) and Fib (mg/dl), AT (%), FDP (µg/ml) concentrations between the different time points. Black lines represent the median values, and the error bars represent the range. (b) Box and whisker plots for the blood clotting system analysis. ACT (seconds) was measured at baseline (0 min), after 5 min of bolus administration (5 min), after 20 min of CRI administration (25 min), and after 30 min of a washout period (55 min). Significant differences were observed between 0 versus 5 min (*p* < 0.05) and 0 versus 25 min (*p* < 0.05). Black lines represent the median values, and the error bars represent the range. * and ** indicate statistical significance at a *p*‐value of <0.05 and < 0.01, respectively. ACT, activated clotting time; APTT, activated thromboplastin time; AT, antithrombin; FDP, fibrin degradation products; Fib, fibrinogen; PT, prothrombin time; CRI, continuous rate infusion.

The median (range) values for haemostasis findings are shown in Table 1. Notably, there was a significant difference in the APTT values at 0 and 25 min (*p* < 0.01; Figure 1a). ACT values were significantly different at 0 versus 5 min (*p* < 0.05) and 0 versus 25 min (*p* < 0.05; Figure 1b). There were no significant differences in PT, Fib, AT and FDP concentrations at any time point.

**TABLE 1:** Blood clotting, complete blood count, and plasma biochemistry results 0 min, 25 min and 55 min after the administration of nafamostat mesilate

	0 min	25 min	55 min
**Blood clotting**			
APTT, s	14.0 (10.0–14.0)	120.0 (40.0–120.0)*	25.5 (23.0–41.0)
PT, s	6.5 (6.0–8.0)	7.0 (7.0–10.0)	7.0 (6.0–8.0)
Fibrinogen, mg/dl	122.5 (106.0–189.0)	137.5 (79.0–174.0)	139.0 (78.0–210.0)
AT, %	130.0 (125.0–133.0)	164.0 (126.0–195.0)	132.5 (111.0–150.0)
FDP, µg/ml	1.9 (0.5–2.4)	1.7 (0.9–1.9)	2.6 (0.5–2.4)
ACT, s	114.0 (86.0–151.0)	982.5** (788.0–1000.0)	158.5** (130.0–219.0)
**Complete blood count**			
WBC, per µl	7300 (6300–8500)	5900** (4000–6600)	7000 (5800–8500)
Haematocrit, %	44.6 (33.9–54.4)	43.7 (35.5–48.8)	44.4 (38.6–50.9)
Platelet, 10⁴/µl	35.4 (26.8–43.2)	37.4 (23.7–43.8)	38.2 (25.9–45.4)
**Plasma biochemistry**			
Total protein, g/dl	5.8 (5.6–6.3)	5.2** (5.0–5.5)	5.4 (4.9–5.7)
Albumin, g/dl	3.0 (2.6–3.2)	2.7** (2.1–2.9)	2.8 (2.1–3.0)
Blood glucose, mg/dl	81.0 (73.0–97.0)	90.0 (65.0–125.0)	86.0 (77.0–216.0)
AST, U/L	31.0 (28.0–61.0)	37.5 (29.0–67.0)	44.0 (31.0–69.0)
ALT, U/L	30.0 (24.0–35.0)	29.0 (22.0–33.0)	34.5 (26.0–49.0)
ALKP, U/L	118.5 (87.0–173.0)	105.0 (78.0–179.0)	106.0 (78.0–192.0)
LDH, U/L	110.5 (78.0–468.0)	192.0 (140.0–493.0)	256.0 (138.0–636.0)
BUN, mg/dl	21.1 (10.2–22.6)	21.3 (10.3–23.1)	20.2 (10.7–24.2)
Creatinine, mg/dl	0.67 (0.63–0.72)	0.69 (0.59–0.73)	0.69 (0.59–0.79)
Sodium, mEq/L	144.5 (144.0–146.0)	145.0 (143.0–147.0)	146.0 (142.0–148.0)
Potassium, mEq/L	4.9 (4.4–5.2)	4.3 (4.0–4.5)	4.0** (3.3–4.5)
Chloride, mEq/L	107.5 (106.0–112.0)	110.0** (108.0–112.0)	108.0 (107.0–111.0)

*Note*: All values are presented as median (range).

* Significant at the *p* < 0.01 level relative to values at 0 min.

** Significant at the *p* < 0.05 level relative to values at 0 min.

ALT, alanine transaminase; ACT, activated clotting time; APTT, activated thromboplastin time; AST, aspartate aminotransferase; AT, antithrombin; BUN, blood urea nitrogen; FDP, fibrin degradation products; LDH, lactate dehydrogenase; PT, prothrombin time; WBC, white blood cell.

### 3.2 Complete blood counts

**FIGURE 2:**
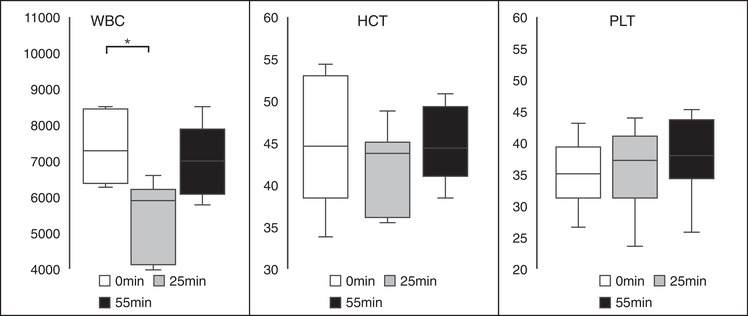
Box and whisker plots of the blood clotting system analysis. The white blood cell (WBC; in per µl) counts at 0, 25, and 55 min are displayed. There were significant differences between 0 versus 25 min (*p* < 0.05); however, there were no significant differences between the other time intervals. There were no significant differences in the haematocrit (%) and platelet (10⁴/µl) counts. The black lines represent the median values and the error bars represent the range. * indicates statistical significance at a *p*‐value of <0.05.

Among the different components of the CBC, only WBC counts differed significantly between 0 and 25 min (*p* < 0.05; Figure 2). Measured CBC parameters for each time point are summarised in Table 1.

### 3.3 Plasma biochemistry

**FIGURE 3:**
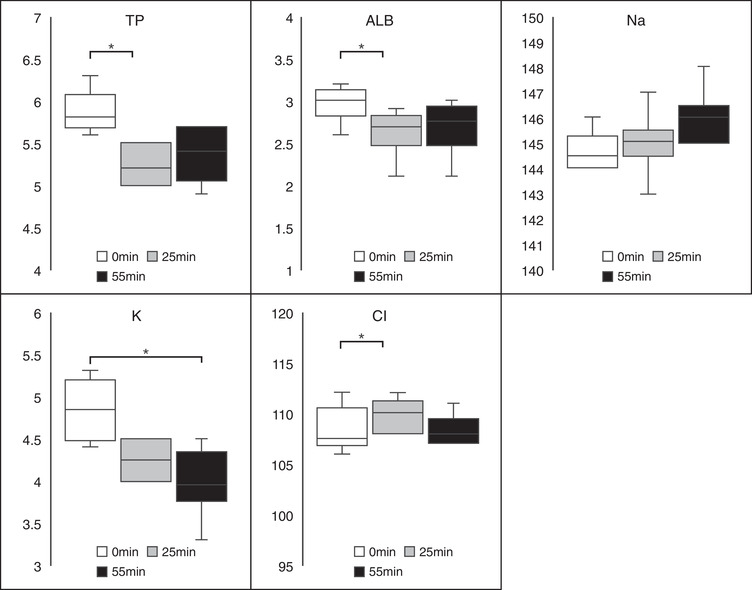
Box and whisker plots of the blood clotting system analysis. Significant differences were observed in the total protein (in g/dl), albumin (g/dl), and chloride (mEq/L) levels between 0 versus 25 min (*p* < 0.05). There were significant differences in the potassium (mEq/L) concentrations between 0 versus 55 min (*p* < 0.05). The black lines represent the median values and the error bars represent the range. *Statistical significance at a *p*‐value of <0.05.

There were several significant differences in plasma biochemistry measurements taken at the 3 time points (Table 1 and Figure 3). A significant difference in protein concentration was observed between 0 and 25 min (*p* < 0.05), albumin concentrations differed significantly between 0 and 25 min (*p* < 0.05), the potassium concentrations were significantly different between 0 and 55 min (*p* < 0.05), and chloride concentrations were significantly different between 0 and 25 min (*p* < 0.05). All other measured biochemistry parameters were not significantly different at any of the time points evaluated in this study.

### 3.4 Blood pressure measurements

**FIGURE 4:**
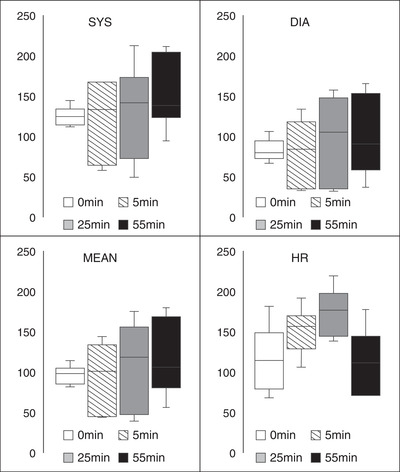
Box and whisker plots of the blood clotting system analysis. The systolic blood pressure (SYS; in mmHg), diastolic blood pressure (DIA; mmHg), mean blood pressure (mmHg) and heart rate (HR; beats per minute). All data were collected at baseline (0 min), after 5 min of bolus administration (5 min), after 20 min of continuous rate infusion administration (25 min) and after a 30 min washout period (55 min). There were no significant differences between any of the parameters investigated at any time period. The black lines represent the median values, and the error bars represent the range.

Table 2 and Figure 4 show the results of the haemodynamic parameters, including systolic blood pressure, diastolic blood pressure, mean blood pressure, and HR, measured at each time point. There were no significant differences in the values of any of these measures at any time point in the study.

**TABLE 2:** Blood pressure and heart rate results 0 min, 5 min, 25 min and 55 min after the administration of nafamostat mesilate

	0 min	5 min	25 min	55 min
**Systolic, mmHg**	124.5 (112.0–144.0)	134.0 (58.0–167.0)	141.0 (50.0–212.0)	138.0 (95.0–210.0)
**Diastolic, mmHg**	79.0 (67.0–106.0)	84.0 (34.0–133.0)	106.0 (33.0–157.0)	89.5 (37.0–165.0)
**Mean, mmHg**	97.5 (82.0–114.0)	100.5 (44.0–114.0)	118.5 (39.0–175.0)	105.5 (57.0–180.0)
**Heart rate, bpm**	114.5 (68.0–181.0)	156.5 (106.0–191.0)	176.0 (138.0–218.0)	112.0 (71.0–177.0)

*Note*: All values are presented as median (range).

### 3.5 Mortality and side effects

All dogs survived the study period. BP changes were observed in four (66.7%) dogs, including high and low BP in two dogs each (33.3% each). No vomiting or diarrhoea was observed during the experiment or after extubation.

## 4 DISCUSSION

This study evaluated the utility and safety of NM use during anaesthesia in dogs. The results obtained from the dogs in this study indicate that APTT was significantly higher at 25 min than at 0 min. No significant differences were observed in the PT, Fib, AT and FDP concentrations. ACT was significantly longer at 5 and 25 min than at 0 min. Blood pressure changes were observed in two‐thirds of the dogs, but the changes were not statistically significant. No other significant differences in CBC and blood chemistry were observed, and no vomiting or diarrhoea was observed during the experiment or after extubation. The data suggest that NM may be safe and efficacious in healthy anaesthetised dogs.

In the current study, APTT and ACT were significantly prolonged after the bolus administration of NM, and both returned to their baseline values after the continuous infusion was discontinued. There were no significant differences between the 0‐ and 55‐min time points in terms of the APTT and ACT. Several studies have used NM in haemodialysis circuits or extracorporeal membrane oxygenation circuits in dogs (Choi et al., 2018; Han et al., 2018; Shimokawa Miyama et al., 2010). However, the administered doses of NM used in these studies were lower than those used in this study. Thus, the ACT did not exceed 300 s, and blood clotting was reported. ACT prolongation for greater than 300 s is required for safe CPB (Bull et al., 1975). Therefore, a bolus administration of NM followed by a high dose of 10 mg/kg/h was used in this study, based on the dosage used in a report that achieved an ACT greater than 300 s in dogs (Okamoto et al., 1993, 1994). NM alone was sufficient to prolong the ACT to achieve the target range in this study. The recovery of the anticoagulant effect was rapid at 30 min after the end of continuous infusion.

Complications such as low BP, tachycardia, hyperkalaemia, and vomiting due to NM use have been reported in the literature (Okajima et al., 1995; Okamoto et al., 1993, 1994; Shimokawa Miyama et al., 2010). There were no significant differences in the BP measurements at different time points, but fluctuations were observed in the dogs with some cases presenting with hypertension and others with hypotension. Moreover, tachycardia was observed during continuous administration. These changes disappeared after the NM administration was completed. There were significant changes in total protein, albumin, potassium and chloride levels and WBC count during the CRI of NM. Additionally, the decrease in potassium levels was observed after the administration was discontinued. These differences were not severe and treatment was not necessary. Additionally, no incidences of vomiting or diarrhoea after NM administration were observed. The potential clinical significance of these changes is unclear and requires further study.

The present study evaluated the anticoagulant effect in normal dogs under anaesthesia; thus, the metabolism of the drug may differ when a high dose is administered during CPB. Therefore, further studies are required during actual CPB to fully evaluate the use of NM during CPB. In particular, prolonged metabolic time may prolong ACT and increase the risk of haemorrhage. Therefore, drug metabolism should be further evaluated under CPB conditions.

The main limitations of this study include the small number of dogs tested, the lack of a control group, limited follow‐up data and limited data on outcomes. The small sample size coupled with the multiple statistical comparisons means that this data may lack sufficient power to detect statistically significant differences. To address this issue, future studies should incorporate larger sample sizes. Although the lack of metabolism assessment is another weakness of our study, it has been reported that there are no serious adverse effects on drug metabolism when NM is administered to dogs for prolonged periods of time, when extracorporeal membrane oxygenation was used or when haemodialysis was used to perform haemodilution (Choi et al. 2018; Han et al., 2018; Okamoto et al., 1993, 1994).

## 5 CONCLUSION

In conclusion, prolongation of the ACT was sufficient when NM was used alone in anaesthetised dogs, and may be safe for use during CPB, although further studies are required. These data should be considered preliminary, and further studies incorporating control groups are required to validate our findings.

## AUTHOR CONTRIBUTIONS

Noriko Isayama: conceptualisation; data curation; writing – original draft; writing – review & editing. Goki Matsumura: conceptualisation; formal analysis; writing – review & editing. Yusuke Uchimura: data curation; writing – review & editing. Erika Maeda: data curation; writing – review & editing. Kenta Sasaki: data curation; writing – review & editing.

## FUNDING STATEMENT

This work was supported by a Grant‐in‐Aid for an Open Research Grant from the Japan Research Promotion Society for Cardiovascular Diseases.

## CONFLICT OF INTEREST

The authors declare that there is no conflict of interest that could be perceived as prejudicing the impartiality of the research reported.

### ETHICAL APPROVAL

The authors confirm that the ethical policies of the journal, as noted on the journal's author guidelines page, have been adhered to, and the appropriate ethical review committee approval has been received. The US National Research Council's guidelines for the Care and Use of Laboratory Animals were followed.

### PEER REVIEW

The peer review history for this article is available at https://publons.com/publon/10.1002/vms3.1002.

## Data Availability

The data that support the findings of this study are available from the corresponding author upon reasonable request.
